# Multigenerational prediction of genetic values using genome-enabled prediction

**DOI:** 10.1371/journal.pone.0210531

**Published:** 2019-01-17

**Authors:** Isabela de Castro Sant’ Anna, Ricardo Augusto Diniz Cabral Ferreira, Moysés Nascimento, Gabi Nunes Silva, Vinicius Quintão Carneiro, Cosme Damião Cruz, Marciane Silva Oliveira, Francyse Edith Chagas

**Affiliations:** 1 Department of Statistics, Federal University of Viçosa, Viçosa, Minas Gerais, Brazil; 2 Syngenta—Seeds Production Research, Uberlândia, Minas Gerais; 3 Department of Statistics, Federal University of Rondônia, Ji-Paraná, Rondônia, Brazil; 4 Department of General Biology, Federal University of Viçosa, Viçosa, Minas Gerais, Brazil; New South Wales Department of Primary Industries, AUSTRALIA

## Abstract

The identification of elite individuals is a critical component of most breeding programs. However, the achievement of this goal is limited by the high cost of phenotyping and experimental research. A significant benefit of genomic selection (GS) to plant breeding is the identification of elite individuals without the need for phenotyping. This study aimed to propose different calibration strategies using combinations between generations from different genetic backgrounds to improve the reliability of GS and to investigate the effects of LD in different types of mating systems: outcrossing (A_n_) self-pollination (S_n_) and hybridization (H_n_). For this purpose, we simulated a genome with 10 linkage groups. In each group, two QTL were simulated. Subsequently, an F_2_ population was created, followed by four generations of inbreeding (S_1_ to S_4,_ H_1_ to H _4,_ A_1_, to A_4,_). Quantitative traits were simulated in three scenarios considering three degrees of dominance (d/a = 0, 0.5 and 1) and two broad sense heritabilities (h^2^ = 0.30 and 0.70), totaling six genetic architectures. To evaluate prediction reliability, a model (RR-BLUP) was trained in one generation and used to predict the following generations of mating systems. For example, the marker effects estimated in the F_2_ population were used to estimate the expected genomic breeding value (GEBV) in populations S_1_ through A_4._ The squared correlation between the GEBV and the true genetic value were used to measure the reliability of the predictions. Independently of the population used to estimate the marker effect, reliability showed the lowest values in the scenario where d = 1. For any scenario, the use of the multigenerational prediction methodology improved the reliability of GS.

## Introduction

Genomic selection (GS) was proposed by [1] as a means to increase selective efficiency, reduce frequency and costs of phenotyping, and also increase annual gains by reducing selection cycle time [2]. This approach, mainly based on the existence of linkage disequilibrium between markers and genes that control traits, employs simultaneous estimation of genetic marker effects that are distributed over the genome in order to explain much of the genetic variation and predict the genetic value of individuals [3].

According to [4] the success of the GS approach depends on the heritability and the genetic architecture of the traits (number and effect of Quantitative trait loci—QTL), as well as on the availability of linkage disequilibrium (LD) between markers and QTL in the training and validation populations [4, 5]. The response to GS relies on linkage disequilibrium (LD), thereby the stronger the LD, the higher the reliability of genomic predictions [6,7,8], and higher the long-term gains[9,10,11,12].

According to [13], the LD between QTL and Single Nucleotide polymorphism (SNP) will decrease over generations and the reliability of genomic prediction is expected to decrease without reestimating the SNP effects in more recent generations. Therefore, estimates obtained to different mating systems, such as outcrossing (allogamous plants), self-pollination (autogamous plants) and hybridization may be affected differently.

In this context, we aimed to (1) propose different calibration strategies using combinations between one or more generations from different genetic backgrounds, here called multigenerational sets, to improve the reliability of GS predictions; (2) to investigate the effects of LD in different types of mating systems (outcrossing, self-pollination and hybridization) on the reliability of GS predictions.

## Material and methods

### Origin of populations

In order to assess the reliability of GS predictions, data were simulated by considering a diploid species with 2n = 2x = 20 chromosomes as the reference, and the total length of the genome was stipulated in 1,000 cM. Genomes were generated with a saturation level of 101 molecular markers spaced by 1cM per linkage group, totaling 1010 markers. Divergent parental line genomes were simulated, as well as genomes from the base population (F_2_), which was used as the reference for obtaining four generations advanced by random mating A_n_ (n = 1,2, 3, 4), self-pollination S_n_ (n = 1,2, 3, 4) and hybridization, represented here by backcrossing involving the F_2_ parent as Bc_n_ (n = 1,2, 3, 4). The effective size of the base population is the size of F_2_ itself, since the base population (F_2_) was derived from two contrasting homozygous parents. This population is in Hardy-Weinberg equilibrium and therefore all disequilibrium is caused by factorial linkage.

### Simulation of quantitative traits

The genotypic value for the monogenic model is defined by u + a, u + d, u–a for the genotypes AA, Aa e aa, respectively. In a polygenic model, the total genotypic value expressed by a given individual belonging to the population was the sum of each additive effects of individual locus estimated by the following expression

$$G_{i}=\mu +a_{i}+d_{i}$$ (1)

where the additive effect (a) of each locus is one half the difference in mean phenotype between the two homozygous genotypes (for each individual i). The dominance effect (d) is the difference between the mean phenotype of the heterozygous genotype and the average phenotype of the two homozygous genotypes. In our simulation we defined 20 loci to control the trait. Therefore, the additive effect is given by:

$$a_{i}=\sum_{j=1}^{20}p_{j}\alpha_{j}$$ (2)

With *α*_*j*_ being the effect of the favorable allele in locus *j*, considered equal to 1, 0 or -1 for the genotypic classes AA, Aa and aa, respectively, and *p*_*j*_ being the contribution of locus *j* to the manifestation of the trait under consideration. In this study, it was established as being equivalent to the probability of the set generated by the binomial distribution (a+b)^s^, where a = b = 0.5 and s = 19. The value of d_i_ was defined according to the average degree of dominance expressed in each trait. The quantitative traits were simulated in three scenarios considering three degrees of dominance (d/a = 0, 0.5 and 1) and two broad sense heritability (h^2^ = 0.30 and 0.70), totaling six genetic architectures.

The phenotypic values of the i^th^ individuals were obtained according to the model: *P*_*i*_ *= G*_*i*_ *+ E*_*i*_, where *G*_*i*_ is the genetic effect given by the sum of the genetic effects in each locus, and *E*_*i*_ is the environmental effect, generated according to a normal distribution with means equal to zero and variance given by the equation bellow:

$$\sigma_{e}^{2}=\frac{\sigma_{g}^{2}(1-h^{2})}{h^{2}}$$ (3)

where $\sigma_{e}^{2}$ is the variance given by the environmental values, $\sigma_{g}^{2}$ is the variance of the genetic values, and *h*^2^ is the heritability defined for the trait. The genetic variance is defined for each population from the information of the genetic control and the importance of each locus in the polygenic model.

$$\sigma_{g}^{2}=\frac{1}{2}{\bar{a}}^{2}+\frac{1}{4}{\bar{d}}^{2},$$ (4)

where ${\bar{a}}^{2},\;{\bar{d}}^{2}$ were defined by the mean values of the effects associated with the homozygote and heterozygous genotypes for each one of the 20 loci, respectively.

### Efficiency of different calibration strategies for GS studies considering different kinds of mating

**Fig 1:**
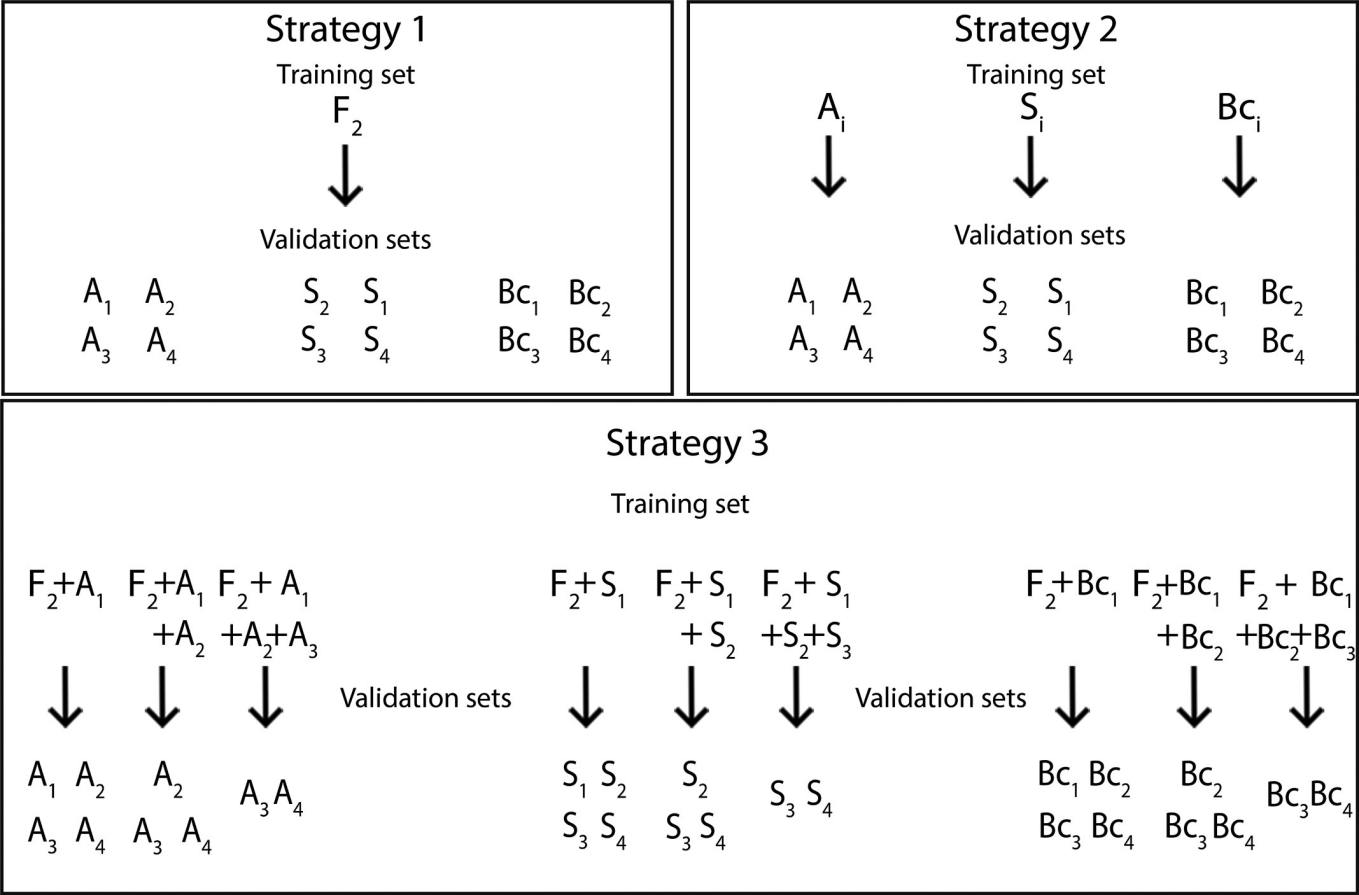
Illustrative scheme of three strategies used in calibration sets of genomic selection training analyses. Letters F_2_, A, S and B_C_ represent the base population of allogamous, self-pollinated and backcrossing species. The index from 1 to 4 represents breeding cycles. In the right square, strategy 2 is the training set composed of species that used the contemporary or outdated genotypic and phenotypic values from the population itself (S_i_, A_i_ and Bc_i_; i = 1,2,3,4), and validation was performed in the same population (S_n_, A_n_ and Bc_n_) and in each advanced population (S_n+1_, A_n+1_ and Bc_n+1_). The last strategy used three different kinds of calibration data sets (D_1t_, D_2t_, D_3t_), which considered multigenerational sets for prediction. In the first training data set (D_1t_), the total of 1000 individuals, 500 from the first generation of allogamous or autogamous species and 500 individuals from the F_2_ population (D_1t_ = A_1_/S_1_+ F_2_) were used to estimate the marker effect. Validation was performed on 500 individuals (D_1v_) from each outdated population (D_1v_ = A_1_/S_1_, A_2_/S_2_, A_3_/S_3_ and A_4_/S_4_). In the second and third kinds of calibration data sets (D_2t_ and D_3t_), data sets were added using generations two (D_2t_ = A_1_/S_1_+ A_2_/S_2_ + F_2_) and three (D_3t_ = A_1_/S_1_+ A_2_/S_2_ + A_3_/S_3_ + F_2_).

In order to assess GS reliability over generations, three calibration strategies were performed. In the first one, we used genotypic and phenotypic information from generation F_2_ for training the model, and validation was performed in each advanced population defined according to the different mating systems. In the second one, the contemporary or outdated genotypic and phenotypic values from each population itself (S_n_, A_n_ and Bc_n_; n = 1,2,3,4) were used, and validation was performed in the same population (S_n_, A_n_ and Bc_n_) and in each advanced population (S_n+1_, A_n+1_ and Bc_n+1_). The last strategy used three different kinds of calibration data sets (D_1t_, D_2t,_ D_3t_), considering multigenerational sets for prediction. In the first training data set (D1t), the total of 1000 individuals, 500 from the first generation of allogamous or autogamous and 500 individuals from the F_2_ population (D_1t_ = A_1_/S_1_+ F_2_), were used to estimate the marker effect. Validation was performed in 500 individuals (D_1v_) from each outdated population (D_1v_ = A_1_/S_1_, A_2_/S_2_, A_3_/S_3_ and A_4_/S_4_). In the second and third kinds of calibration data sets (D_2t_ and D3t), the sets were added using generations two (D_2t_ = A_1_/S_1_+ A_2_/S_2_ + F_2_) and three (D_3t_ = A_1_/S_1_+ A_2_/S_2_ + A_3_/S_3_ + F_2_). These models were, respectively, validated using the true breeding values (TBV) of the population itself (S_n_, A_n_) and in each advanced population (S_n+1_ and A_n+1_) (Fig 1).

The additive dominance model for the REML/G-BLUP method is given by [1]:

$$y=X_{b}+Zu_{a}+Zu_{d}+e,$$ (5)

where *y* is the vector of phenotypic observations, *b* is the vector of fixed effects, *u*_*a*_ is the vector of random of additive marker effects, *u*_*d*_ is the vector of random of dominance marker effects and *e* refers to the vector of random errors; The variance structure is given by *u*_*a*_~*N(0*, *G*_*a*_$\sigma_{u_{a}}^{2}$*); u*_*d*_~*N(0*, *G*_*d*_$\sigma_{u_{d}}^{2}$*); by e*~*N(0,* $I\sigma_{e}^{2}$*)*.

An equivalent model at the marker level is given by

$$y=Xb+ZWm_{a}+ZSm_{d}+e,$$ (6)

where: *u*_*a*_ = *Wm*_*a*_;*Var(Wm*_*a*_*)* = $WI\sigma_{m_{a}}^{2}$ ${W’=WW’\sigma }_{m_{a}}^{2}$*; u*_*d*_ = *Sm*_*d*_; ${Var(Sm}_{d})=SI\sigma_{m_{d}}^{2}S’=SS’\sigma_{m_{a}}^{2}$;

***X*** and ***Z*** are matrices of incidence for the vectors additive (*ma) and dominance* (*m*_*d*_*) marker genetic effects*. The variance components associated to these effects are $\sigma_{m_{a}}^{2}$ and $\sigma_{m_{d}}^{2}$, respectively. *G*_*a*_ and *G*_*d*_ are the genomic relationship matrices for the additive and dominance effects. The quantity *m*_*a*_ in one locus is the allele substitution effect and is given by *m*_*a*_ = α_*i*_ = *a*_*i*_ + (*q*_*i*_ − *p*_*i*_)*di*, where *p*_*i*_ and *q*_*i*_ are allelic frequencies and *a*_*i*_ and d_*i*_ are the genotypic values for one homozygote and heterozygote, respectively, at locus *i*. In turn, the quantity *m*_*d*_ can be directly defined as *m*_*di*_ = *d*_*i*_. The matrices ***W*** and ***S*** are defined based on the values 0, 1 and 2 for the number of one of the alleles at the *i* marker locus in a diploid individual. The correct parameterization of ***W*** and ***S*** is as follows, according to the marker genotypes at a locus m.

$$W=\left\{ \begin{array}{c} MM:2-2p\to 2q\\ Mm:1-2p\to q-p\\ mm:2-2p\to 2q \end{array}\right.$$

$$S=\left\{ \begin{array}{c} MM:0\to -{2q}^{2}\\ Mm:1\to 2pq\\ mm:0\to -{2p}^{2} \end{array}\right.$$

The covariance matrix for the additive effects is given by $G_{a}\sigma_{a}^{2}=Var(Wm_{a})={WW^{\prime }\sigma }_{m_{a}}^{2}$ = ) = , which leads to: $G_{a}=WW^{\prime }/(\sigma_{m_{a}}^{2}/\sigma_{a}^{2})=WW^{\prime }/\sum_{i=1}^{n}\left[{2p}_{i}(1-p_{i})\right]$, as $\sigma_{a}^{2}=\sum_{i=1}^{n}[{2p}_{i}(1-p_{i})]\sigma_{m_{a}}^{2}$. The covariance matrix for the dominance effects is given by $G_{d}\sigma_{d}^{2}=Var(Sm_{d})\;{{SS}^{\prime }\sigma }_{m_{d}}^{2}$. Thus , $G_{d}=SS^{\prime }/(\sigma_{m_{d}}^{2}/\sigma_{d}^{2})={SS}^{\prime }/\sum_{i=1}^{n}{\left[{2p}_{i}(1-p_{i})\right]}^{2}$ as $\sigma_{d}^{2}=\sum_{i=1}^{n}{\left[{2p}_{i}(1-p_{i})\right]}^{2}\sigma_{m_{d}}^{2}$

The additive-dominance G-BLUP method was fitted using Genomic Land software [18] via REML through mixed model equations.

After obtaining the GEBVs, reliability, which is defined by the square correlation between the genomic estimated breeding values and the true breeding values (TBV), was estimated [9].

Finally, linkage disequilibrium was estimated using the maximum likelihood method [14,15]. It was chosen to represent the disequilibrium squared correlation coefficient between two loci *r*^2^ [16] for pairs of loci. The LD measure, *r*^2^, corresponds to the ratio of genetic variance controlled by the QTL whose associated marker is able to explain.

### Computational applications for data analysis

The simulations were implemented with software GENES [17]) and the statistical analyses were performed with software Genomic Land [18].

## Results

### Studying LD in all populations

**Fig 2:**
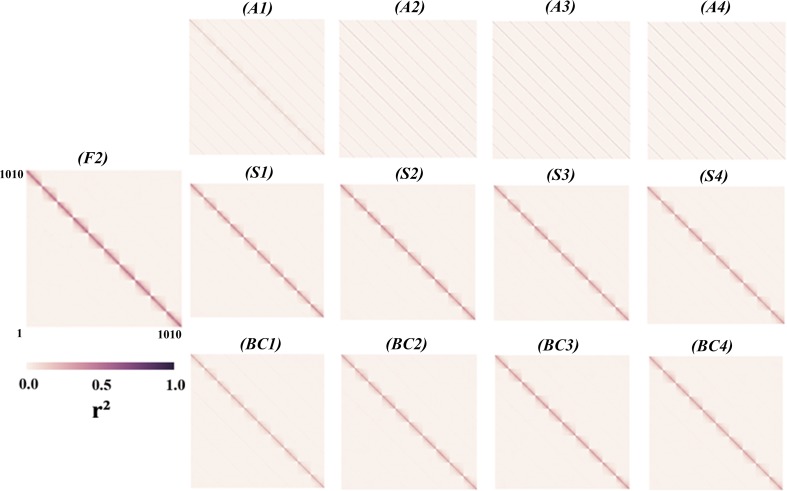
From left to right, estimation of Linkage Disequilibrium measured by r^2^ statistic in F_2_, allogamous set (top), autogamous set (center), and backcrossing set (bottom). X and Y axes represent the (1010) markers distributed in 10 linkage groups with 101markers each. The intensite of correlation inside each linkage block decreased the as we observe advanced generations. These results are represented in scatter plots of r^2^ values versus the genetic or physical distance between all pair of alleles (Fig 2).

Linkage disequilibrium (*r*^2^) was calculated for all pairwise physical distances among all the SNPs (in each linkage group separately. The average genome-wide LD for pairs of SNPs within a 1 centimorgan distance from each population group was 0.006 for allogamous species, 0.051 for autogamous species, 0.037 for hybridization, and 0.189 for F_2_. These results are represented in scatter plots of r^2^ values versus the genetic or physical distance between all pair of alleles (Fig 2). The linkage disequilibrium was almost entirely dissipated in allogamous population since the first generation but continues in autogamous and backcrossing sets.

### Calibration using the F_2_ population

We contrasted the reliability of the linear models (G-BLUP) considering scenarios with and without dominance in different levels of heritability over generations. Overall, the reliability values decreased over generations for all the scenarios considered. Specifically, for the scenarios without dominance (d = 0) and low heritability (h^2^ = 0.30), the averages of reliability values were 0.55, 0.20 and 0.50 for, respectively, outcrossing (allogamous species), selfing-pollination (autogamous species) and hybridization mating systems (Table 1). In the scenarios with dominance (d = 0.5 and d = 1) and low heritability, the averages of reliability values changed from 0.49, 0.15, 0.44 to 0.48, 0.17 and 0.43, respectively, for outcrossing (allogamous species), self-pollination (autogamous species) and hybridization mating systems. The GS reliability considering the F_2_ population as training and test population was equal to 0.81, 0.72 and 0.70 for the scenarios with low heritability and, respectively, no dominance, partial (d = 0.5) and complete (d = 1) dominance. The results observed for the high heritability scenarios (h^2^ = 0.70) were similar when compared with those obtained for the low heritability scenarios (Table 1).

**Table 1:** Reliability values of selection of generations advanced by self-pollination (S_n_), outcrossing (A_n_) and hybridization (Bc_n_) (obtained from phenotyping and genotyping of the F_2_ population for six traits (heritability equal to 0.30 and 0.70 repeated in scenarios with an average degree of dominance(d) level equal to 0, 0.5 and 1). The scale of colors changes from 0 (red) to 0.5 (yellow) to 1(green).

**Low Heritability**																
**d**	**F**_**2**_	**S**_**1**_	**S**_**2**_	**S**_**3**_	**S**_**4**_	**Mean**	**A**_**1**_	**A**_**2**_	**A**_**3**_	**A**_**4**_	**Mean**	**Bc**_**1**_	**Bc**_**2**_	**Bc**_**3**_	**Bc**_**4**_	**Mean**
**0**	0.81± 0.03	0.63 ± 0.07	0.56 ± 0.05	0.53 ± 0.06	0.51 ± 0.04	0.55	0.31 ± 0.06	0.22 ± 0.03	0.16 ± 0.02	0.11 ± 0.01	0.20	0.45 ± 0.01	0.50 ± 0.05	0.53 ± 0.05	0.52 ± 0.04	0.50
**0.5**	0.72 ± 0.03	0.55 ± 0.01	0.49 ± 0.00	0.47 ± 0.00	0.46 ± 0.01	0.49	0.23 ± 0.03	0.18 ± 0.01	0.12 ± 0.00	0.08 ± 0.01	0.15	0.40 ± 0.01	0.42 ± 0.02	0.47 ± 0.01	0.46 ± 0.01	0.44
**1**	0.70 ± 0.10	0.51 ± 0.08	0.48 ± 0.06	0.47 ± 0.07	0.47 ± 0.04	0.48	0.20 ± 0.03	0.20 ± 0.04	0.15 ± 0.03	0.12 ± 0.04	0.17	0.36 ± 0.07	0.41 ± 0.05	0.49 ± 0.07	0.47 ± 0.09	0.43
**High Heritability**																
**0**	0.94 ± 0.01	0.73 ± 0.01	0.65 ± 0.01	0.62 ± 0.01	0.60 ± 0.00	0.65	0.39 ± 0.01	0.28 ± 0.02	0.19 ± 0.02	0.14 ± 0.02	0.25	0.52 ± 0.00	0.57 ± 0.01	0.62 ± 0.01	0.62 ± 0.00	0.58
**0.5**	0.89 ± 0.05	0.69 ± 0.04	0.62 ± 0.04	0.60 ± 0.04	0.59 ± 0.04	0.68	0.35 ± 0.05	0.28 ± 0.07	0.21 ± 0.05	0.15 ± 0.08	0.25	0.48 ± 0.03	0.51 ± 0.04	0.59 ± 0.04	0.57 ± 0.05	0.54
**1**	0.89 ± 0.01	0.65 ± 0.02	0.58 ± 0.00	0.58 ± 0.01	0.57 ± 0.00	0.65	0.23 ± 0.02	0.22 ± 0.01	0.16 ± 0.03	0.11 ± 0.00	0.18	0.46 ± 0.01	0.51 ± 0.01	0.58 ± 0.01	0.57 ± 0.00	0.52

### Calibration using contemporary or outdated populations

Overall, the second calibration strategy outperforms the results obtained with the first calibration strategy, which used only the F_2_ population for calibration. For example, population A_4_ had the worst reliability prediction value, 0.08 (Table 1), using training strategy 1, and improved to 0.49 (Table 2) using training strategy 2. The reliability values for the different mating systems (outcrossing—allogamous, self-pollination-autogamous and hybridization-backcrossing) obtained using contemporary or outdated populations are displayed in Table 2. Overall, for the scenarios with lower heritability, the inclusion of outdated calibration sets allowed to increase the average of reliability values in allogamous population and maintain in the others mating systems. Specifically, the averages for the allogamous populations (A_1_ to A_4_), in the scenario with low heritability, increased from 0.20, 0.15, 0.17 to 0.42, 0.40, 0.34, for, respectively, the scenarios without dominance (d = 0), and those with dominance equal to 0.5 and 1.

**Table 2:** Reliability values of selection of generations advanced by self-pollination(S_n_), random mating (A_n_) and hybridization (Bc_n_) obtained from phenotyping and genotyping of the same generation (diagonally contemporary) or only from previous genotyping and phenotyping (outdated off-diagonally, horizontal reading) for the heritability trait equal to 0.30 in the scenario with an average degree of dominance equal to 0, 0.5 and 1.

						Validation set							
Traits	Training set	S_1_	S_2_	S_3_	S_4_	A_1_	A_2_	A_3_	A_4_	Bc_1_	Bc_2_	Bc_3_	Bc_4_
d0	S_1_	0.61 ± 0.06	0.55± 0.10	0.53 ± 0.10	0.52 ± 0.10								
	S_2_		0.54 ± 0.09	0.53 ± 0.10	0.54 ± 0.10								
	S_3_			0.54 ± 0.10	0.60 ± 0.10								
	S_4_				0.59 ± 0.02								
	A_1_					0.42 ± 0.02	0.37 ± 0.03	0.32 ± 0.01	0.33 ± 0.03				
	A_2_						0.44 ± 0.12	0.40 ± 0.10	0.40 ± 0.12				
	A_3_							0.50 ± 0.12	0.53 ± 0.09				
	A_4_								0.46 ± 0.11				
	Bc_1_									0.30 ± 0.09	0.34 ± 0.01	0.36 ± 0.02	0.36 ± 0.02
	Bc_2_										0.55 ± 0.05	0.55 ± 0.06	0.54 ± 0.08
	Bc_3_											0.55 ± 0.05	0.51 ± 0.04
	Bc_4_												0.52 ± 0.07
	Average set	0.54 ± 0.07				0.42 ± 0.08				0.46 ± 0.05			
						Validation set							
Traits	Training set	S_1_	S_2_	S_3_	S_4_	A_1_	A_2_	A_3_	A_4_	Bc_1_	Bc_2_	Bc_3_	Bc_4_
d0.5	S_1_	0.61 ± 0.11	0.52 ± 0.03	0.48 ± 0.00	0.46 ± 0.00								
	S_2_		0.55 ± 0.13	0.54 ± 0.08	0.53 ± 0.08								
	S_3_			0.60 ± 0.10	0.54± 0.07								
	S_4_				0.55± 0.07								
	A_1_					0.48± 0.10	0.36 ± 0.03	0.34 ± 0.07	0.33± 0.11				
	A_2_						0.44 ± 0.10	0.35 ± 0.11	0.34 ± 0.11				
	A_3_							0.48 ± 0.10	0.40 ± 0.01				
	A_4_								0.49 ± 0.12				
	Bc_1_									0.53 ± 0.14	0.47 ± 0.15	0.49 ± 0.15	0.47 ± 0.11
	Bc_2_										0.50 ± 0.14	0.51 ± 0.09	0.48 ± 0.11
	Bc_3_											0.55 ± 0.10	0.48 ± 0.06
	Bc_4_												0.55 ± 0.10
	Average set	0.53 ± 0.06				0.40± 0.10				0.50 ± 0.10			
						Validation set							
Traits	Training set	S_1_	S_2_	S_3_	S_4_	A_1_	A_2_	A_3_	A_4_	Bc_1_	Bc_2_	Bc_3_	Bc_4_
d1	S_1_	0.51 ± 0.08	0.49± 0.09	0.49 ± 0.10	0.49± 0.08								
	S_2_		0.53 ± 0.10	0.53 ± 0.09	0.52 ± 0.08								
	S_3_			0.56 ± 0.07	0.56± 0.11								
	S_4_				0.55± 0.08								
	A_1_					0.41 ± 0.11	0.27 ± 0.10	0.27 ± 0.10	0.26 ± 0.11				
	A_2_						0.39 ± 0.11	0.29± 0.10	0.24 ± 0.10				
	A_3_							0.48 ± 0.11	0.36 ± 0.07				
	A_4_								0.47 ± 0.09				
	Bc_1_									0.49 ± 0.11	0.39 ± 0.03	0.44 ± 0.05	0.40 ± 0.05
	Bc_2_										0.54 ± 0.10	0.46± 0.07	0.45 ± 0.08
	Bc_3_											0.45± 0.11	0.38± 0.08
	Bc_4_												0.53 ± 0.06
	Average set	0.52 ± 0.11				0.34 ± 0.07				0.45 ± 0.09			

For the autogamous population (S_1_ to S_4_), the averages of reliability values previously obtained with strategy 1 were 0.55 (d = 0), 0.49 (d = 0.5) and 0.48 (d = 1) and increased to 0.54 (d = 0), 0.53 (d = 0.5) and 0.52(d = 1) using strategy 2. For the hybridization set (Bc_1_ to Bc_4_), GS reliability was 0.50(d = 0), 0.41 (d = 0.5) and 0.43 (d = 1) with strategy 1, staying around 0.46 (d = 0), 0.50 (d = 0.5) and 0.45(d = 1) with strategy 2. The results for the scenarios with higher heritability show same pattern that those observed for scenarios with low heritability (h^2^ = 0.30). For higher heritability scenarios (h^2^ = 0.70), the calibration strategy 2 outperform the results obtained from strategy 1 for all mating systems (S1 Table).

### Calibration using multigenerational populations

The reliability values considering the utilization of combined generations in the calibration set, here called multigenerational sets, for all different scenarios, are presented in Table 3. This strategy increased the reliability values in all populations assessed for the scenarios with low heritability in the three degrees of dominance. For the scenarios with no dominance, the average of reliability values increased from 0.20 (strategy 1) to 0.42 (strategy 2) and 0.63 (strategy 3) in outcrossing (allogamous) populations. In self-pollination (autogamous) and hybridization (backcrossing) populations, the averages of values increased from 0.54 (strategies 1 and 2) to 0.64 (strategy 3), and the reliability values increased from 0.50 (strategy 1) to 0.71 (strategy 3), although decreased to 0.46 (strategy 2).

**Table 3:** Reliability values of selection of generations advanced by self-pollination (S_n_) random mating (A_n_) and hibridization (Bc_n_) obtained from phenotyping and genotyping of combined previous generations (multigenerational) or only from previous genotyping and phenotyping for heritability traits equal to 0.30, repeated in scenarios with an average degree of dominance level equal to 0, 0.5 and 1.

dominance	0				0.5				1			
Multigerational Training	S_1_	S_2_	S_3_	S_4_	S_1_	S_2_	S_3_	S_4_	S_1_	S_2_	S_3_	S_4_
F_2_S_1_	0.64 ± 0.10	0.62 ± 0.10	0.60 ± 0.10	0.59 ± 0.08	0.65 ± 0.06	0.58 ± 0.06	0.57 ± 0.06	0.55 ± 0.06	0.61 ± 0.07	0.57 ± 0.06	0.56 ± 0.07	0.56 ± 0.04
F_2_S_1_S_2_		0.66 ± 0.11	0.65 ± 0.10	0.64 ± 0.09		0.65 ± 0.07	0.63 ± 0.06	0.63 ± 0.05		0.64 ± 0.08	0.63 ± 0.07	0.62 ± 0.05
F_2_S_1_S_2_S_3_			0.71 ± 0.08	0.69 ± 0.08			0.71 ± 0.08	0.70 ± 0.07			0.69 ± 0.11	0.68 ± 0.09
Average Set	0.64 ± 0.07				0.64 ± 0.06				0.63 ± 0.07			
Multigerational Training	A_1_	A_2_	A_3_	A_4_	A_1_	A_2_	A_3_	A_4_	A_1_	A_2_	A_3_	A_4_
F_2_A_1_	0.61 ± 0.10	0.64 ± 0.10	0.57 ± 0.10	0.62 ± 0.10	0.53 ± 0.09	0.39 ± 0.03	0.38 ± 0.08	0.36 ± 0.11	0.48 ± 0.10	0.37± 0.06	0.36± 0.06	0.36 ± 0.10
F_2_A_1_A_2_		0.62 ± 0.10	0.63 ± 0.10	0.64 ± 0.10		0.55 ± 0.10	0.50 ± 0.10	0.47± 0.10		0.51 ± 0.10	0.44 ± 0.10	0.43 ± 0.10
F_2_A_1_A_2_A_3_			0.68 ± 0.08	0.68 ± 0.10			0.63 ± 0.10	0.58 ± 0.08			0.60 ± 0.09	0.53± 0.11
Average Set	0.63 ± 0.10				0.48 ± 0.11				0.45 ± 0.10			
Multigerational Training	Bc_1_	Bc_2_	Bc_3_	Bc_4_	Bc_1_	Bc_2_	Bc_3_	Bc_4_	Bc_1_	Bc_2_	Bc_3_	Bc_4_
F_2_Bc_1_	0.71 ± 0.04	0.67 ± 0.01	0.68 ± 0.01	0.67 ± 0.00	0.61 ± 0.05	0.55 ± 0.02	0.58 ± 0.03	0.57 ± 0.00	0.54 ± 0.10	0.47 ± 0.10	0.54 ± 0.09	0.51 ± 0.10
F_2_Bc_1_Bc_2_		0.75 ± 0.02	0.73 ± 0.01	0.70 ± 0.01		0.66 ± 0.07	0.65 ± 0.03	0.62 ± 0.01		0.60 ± 0.10	0.59 ± 0.06	0.55± 0.08
F_2_Bc_1_Bc_2_Bc_3_			0.79 ± 0.02	0.73 ± 0.01			0.71 ± 0.06	0.66 ± 0.02			0.65 ± 0.07	0.58 ± 0.07
Average Set	0.71 ± 0.02				0.62 ± 0.02					0.55 ± 0.02		

In the scenarios with dominance (d = 0.5), the average of reliability values increased from 0.15(strategy 1) to 0.40 (strategy 2) and 0.48(strategy 3) in allogamous populations. In autogamous and hybridization populations, the average of values increased from 0.49(strategies 1), and 2) to 0.53 (strategy 2) to 0.64 (strategy 3), and the reliability values increased from 0.41 (strategy 1) to 0.50 (strategy 2) to 0.62 (strategy 3).

In the scenarios with dominance (d = 1), the averages of reliability values increased from 0.17 (strategy 1) to 0.34 (strategy 2) and 0.45 (strategy 3) in allogamous populations. In autogamous and hybridization populations, the averages of values were 0.48(strategy 1), 0.52 (strategy 2) and 0.63 (strategy 3), and the reliability values increased from 0.43 (strategy 1) to 0.45 (strategy 2) 0.55 (strategy 3). The results for the higher heritability scenarios (0.7) follow the same pattern of reliability values. It can be seen in supplementary material (S2 Table).

For the group of allogamous populations, the inclusion of only the first random-mating generation enabled the averages of reliability values to around 0.50 within d = 0.5 or without dominance. The inclusion of phenotypic data with advanced generations as 2 or 3 previous generations (F_2_, A_1_, A_2_, for example) improved even more the reliability values in the advanced generations A_3_ and A_4_. In the autogamous populations, the inclusion of two or more previous generations in the training allowed obtaining reliability above 0.61 in all three scenarios, as showed in Table 3. For the hybridization strategy 3 increased the reliability values to above 0.45 within or without dominance. Overall, the inclusion of only one more generation with the F_2_ population sufficed to increase the prediction process over all scenarios.

## Discussion

Our study was designed to maximize GS reliability in terms of the available genetic material. In contrast to previous studies on GS, we do not compare different prediction methodologies, such as G-BLUP, and different Bayesian approaches. In this study, we investigate different calibration strategies using combinations between one or more generations from different genetic backgrounds to improve the reliability of GS predictions. Furthermore, we investigated the effects of LD in different types of mating systems on the reliability of GS predictions. We used the G-BLUP on simulated data sets to access the reliability values for all evaluated scenarios.

### Studying LD in all populations

In the F_2_ population, the linkage disequilibrium was, solely, provided by the factorial linkage with the formation of 10 blocks of disequilibrium corresponding to the 10 linkage groups simulated for the genome (Fig 2). These factorial linkage effects on disequilibrium occur in different ways in successive generations of outcrossing or self-pollination. For example, in allogamous populations and with the progression of generations, it was possible to notice that disequilibrium was almost entirely dissipated, probably leading to low efficiency of procedures, such as GS, whose principle was the existence of disequilibrium between the marker and genes of interest.

According to the study of linkage disequilibrium carried out by Sorkheh and Malysheva-Otto (2008), random mating is an impactful factor in the decrease of linkage disequilibrium over time. Thus, conservation of linkage disequilibrium values in autogamous and backcrossing populations is justified by the fact that, for an effective recombination, double heterozygotes are required, and these are much more common in allogamous than in autogamous species. In addition, for autogamous species, linkage disequilibrium extends over large physical distances in comparison to outcrossing species, as verified by [19,20,21,22,23,24] which justifies the fact that the means of LD among all pairs of loci is 0.006 for allogamous and 0.051 for autogamous species. We believed that the impact of random mating on linkage disequilibrium can justify the importance of outdated calibration sets in allogamous populations.

### Calibration using the F_2_ population

We analyzed the reliability values for all the populations trained using F_2_ in scenarios in which heritability were equal to 0.3 and 0.7 with and without dominance. In this analysis, we sought to emphasize some important issues. The first one refers to the extent to which disturbing factors, such as dominance and environmental noise (influencing character heritability), can affect the reliability of the selection process via GS within the F_2_ generation itself. However, the most important issue is the extent to which the GS efficacy is compromised when one goes from one generation to another where the loss of disequilibrium will be manifested, as such a loss is more intense in random-mating generations than in those from hybridization and self-pollination. These results suggest that the type of crossing shows a major contribution to the low reliability values.

Overall the presence of dominance also contributes to decrease in the prediction process, which is aggravated in the outcrossing populations in all three strategies. Almeida Filho [25], who simulated a genetically similar population of loblolly pine to assess the predicting ability of polygenic and oligogenic traits controlled by different degrees of dominance, also observed a drop in the prediction process reliability across generations. Their study showed that prediction in subsequent progeny population improved only in dominance superior to 0.2 and for oligogenic traits. In our study, genomic prediction reliability the assessment encompassed traits measured in the 12 populations that were previously genotyped and phenotyped for each trait. As displayed in Table 1, the reliability of the predictions increased when we changed heritability from 0.3 to 0.7. However, when we increased dominance from 0 to 1, the values decreased in the majority of scenarios. For example, in particular cases with allogamous species, the scenario with heritability 0.7 and dominance equal to 1 the prediction values were three times lower than those obtained for self-pollination and hybridization (0.65 and 0.52). This is expected because we know that dominance is a disturbing factor, since even when dominance is including in the model the reliability depends on considering heterosis and future combinations between parents. In general, the results showed a decrease in the reliability of total genomic predictions when the dominance increased. Although dominance was contemplated in the linear model used, according to some authors [4, 25, 26, 27], its incorporation does not lead to an improvement in the prediction process reliability of complex traits. In addition, according to Toro and Varona [28] when working with polygenic traits in simulated populations, the additive-dominant models exceeded the additive models only in the first predictive generation. Therefore, all these authors concluded that dominance increases in terms of complexity of the models, but it does not increase in terms of reliability.

### Efficiency of different calibration strategies for GS studies considering different kinds of mating

The drop in reliability observed for the predictions of successive generations of random mating, hybridization or self-pollination is expected not only because of the drop in the disequilibrium of subsequent generations, but also because of the lack of re-estimation of markers, i.e., a new genotyping. According to [29], if genomic selection is practiced for many generations the effect of the markers does not change but the proportion of the genetic variance explained by them declines. This will cause the rate of response to selection to decline, as found by[13] using simulation. Thus, the inclusion of the latest genotyping, i.e., of genotyped and phenotyped individuals in their own generation, leads to an increase in the reliability estimates and can even be used in the prediction of subsequent generations with the same population structure, as displayed in Table 3. According to [30], this occurs because, despite the useful feature of genomic selection that long term response is predictable as the marker allele frequencies are known, it ignores non-additive effects of the QTL which may cause a change in the gene substitution effect of the QTL and, therefore, in the apparent effect of the marker, as selection changes gene frequencies. Several authors change the index weights as selection proceeds[29,30,31]. Naturally, it would be possible to continually re-estimate the marker effect and include additional markers–those that had been excluded in the initial index. In our case, we demonstrated, by using a heritability of 0.3, that investment in genotyping of a new generation is important to ensure the efficiency of the prediction process, especially in populations whose linkage disequilibrium had already disappeared, as observed in our study, as well as for the fourth generation of population structures used here. For instance [11]predicted the breeding value of descendants 10 generations after the generation in which the phenotypes were recorded. Consequently, the reliability of GEBVs, calculated using the relationship matrix derived from the pedigree, was close to zero, so the reliability of the GEBVs using marker data was independent of the pedigree relationships.

#### The best strategy for prediction in allogamous populations

The importance of population structures (allogamous, autogamous, and hybridization systems) was also investigated to allow inferring whether training and validation could be performed with different genetic backgrounds. Thus, considering a random-mating population, we observed that reliability is reduced with the progress of generations by loss of disequilibrium. However, this reduction may be reverted if information from other generations of populations with the same structure is added to the training process, thus increasing reliability we used here in strategy 3. Therefore, especially for allogamous species, population structure is more impactful than linkage disequilibrium [32].

These results were also observed by [33] who conducted their study using one set of 73,147 marker in rice to predict five traits in the 2012 dry and wet seasons. Their study showed that the level of family relationship between selection candidates and the reference population had a higher effect on the reliability of genomic values than on linkage disequilibrium *per se*. Similar results were also found by [11], who, by using a simulation with genes in equilibrium, found that the prediction effects of selection were not zero because the family relationships were important, even in the absence of linkage disequilibrium.

Depending on the family structure of the data, there may also be information on the within-family marker effect, as obtained from a linkage analysis, and this may also add to the reliability of the GEBVs. Hence, in practical terms, one can assume that, in allogamous species, the prediction of advanced generations from early training may not be advantageous due to the loss of disequilibrium, which according to [5], for any pair of linked polymorphic loci, LD decreases over generations because of accumulation of recombination. Consequently, the most interesting solution is a new, more contemporary genotyping combined with the information from a previous genotyping (Strategy 3). A more evident example is that in scenarios with a high dominance degree (d/a = 1), the inclusion of F_2_ and the three generations can better predict A_4_ than only F_2_ and A_1_. Thus, it seems that the population structure becomes even more important in scenarios of high dominance.

## Conclusion

As a result, we improved the reliability values of all the scenarios tested with high complexity. We highlight the importance of calibrating the training model used in GS in order to improve prediction in scenarios with low heritability and high dominance.

## Supporting information

S1 Table Reliability values of selection of generations advanced by self-pollination(S_n_), random mating (A_n_) and hybridization (Bc_n_) obtained from phenotyping and genotyping of the same generation (diagonally contemporary) or only from previous genotyping and phenotyping (outdated off-diagonally, horizontal reading) for the heritability trait equal to 0.30 in the scenario with an average degree of dominance equal to 0, 0.5 and 1. (DOCX)

S2 Table Reliability values of selection of generations advanced by self-pollination (S_n_) random mating (A_n_) and hibridization (Bc_n_) obtained from phenotyping and genotyping of combined previous generations (multigenerational) or only from previous genotyping and phenotyping for heritability traits equal to 0.70, repeated in scenarios with an average degree of dominance level equal to 0, 0.5 and 1. (DOCX)
