# Supplementary material for: Multigenerational prediction of genetic values using genome-enabled prediction
Source: PLoS One. 2019 Jan 17;14(1):e0210531. doi: 10.1371/journal.pone.0210531 (PMC6336252; doi:10.1371/journal.pone.0210531)
Supplement: S1 Table — (DOCX) [file pone.0210531.s001.docx]

|  | **VALIDATION SET** | | | | | | | | | | | | | | |
| --- | --- | --- | --- | --- | --- | --- | --- | --- | --- | --- | --- | --- | --- | --- | --- |
| Trait | **Training** | | **S_1_** | **S_2_** | **S_3_** | **S_4_** | | **A_1_** | **A_2_** | **A_3_** | **A_4_** | **Bc_1_** | **Bc_2_** | **Bc_3_** | **Bc_4_** |
| d0 | **S_1_** | **0.73 ± 0.01** | | **0.65 ± 0.01** | **0.62 ± 0.01** | **0.60 0.00** | |  |  |  |  |  |  |  |  |
|  | **S_2_** |  | | **0.82 ± 0.00** | **0.80 ± 0.00** | **0.79 ± 0.00** | |  |  |  |  |  |  |  |  |
|  | **S_3_** |  | |  | **0.84 ± 0.00** | **0.81 ± 0.01** | |  |  |  |  |  |  |  |  |
|  | **S_4_** |  | |  |  | **0.86 ± 0.00** | |  |  |  |  |  |  |  |  |
|  | **A_1_** |  | |  |  |  | | **0.62 ± 0.02** | **0.60 ± 0.02** | **0.58 ± 0.01** | **0.59 ± 0.00** |  |  |  |  |
|  | **A_2_** |  | |  |  |  | |  | **0.74 ± 0.01** | **0.73 ± 0.00** | **0.74 ± 0.01** |  |  |  |  |
|  | **A_3_** |  | |  |  |  | |  |  | **0.80 ± 0.00** | **0.81 ± 0.01** |  |  |  |  |
|  | **A_4_** |  | |  |  |  | |  |  |  | **0.82 ± 0.01** |  |  |  |  |
|  | **Bc_1_** |  | |  |  |  | |  |  |  |  | **0.46 ± 0.02** | **0.48 ± 0.00** | **0.49 ± 0.00** | **0.46 ± 0.01** |
|  | **Bc_2_** |  | |  |  |  | |  |  |  |  |  | **0.77 ± 0.01** | **0.76 ± 0.01** | **0.60 ± 0.10** |
|  | **Bc_3_** |  | |  |  |  | |  |  |  |  |  |  | **0.77 ± 0.02** | **0.56 ± 0.10** |
|  | **Bc_4_** |  | |  |  |  | |  |  |  |  |  |  |  | **0.60 ± 0.10** |
|  | **Average Set** | **0.75 ± 0.00** | | | | | | **0.70 ± 0.01** | | | | **0.60 ± 0.06** | | | |
|  |  |  | |  |  |  | | **Validation set** |  |  |  |  |  |  |  |
| Traits | **Tranning set** | **S_1_** | | **S_2_** | **S_3_** | **S_4_** | | **A_1_** | **A_2_** | **A_3_** | **A_4_** | **Bc_1_** | **Bc_2_** | **Bc_3_** | **Bc_4_** |
| d0.5 | **S_1_** | **0.76 ± 0.12** | | **0.64 ± 0.06** | **0.60 ± 0.04** | **0.58 ± 0.04** | |  |  |  |  |  |  |  |  |
|  | **S_2_** |  | | **0.78 ± 0.09** | **0.74 ± 0.06** | **0.73 ± 0.06** | |  |  |  |  |  |  |  |  |
|  | **S_3_** |  | |  | **0.82 ± 0.05** | **0.81 ± 0.05** | |  |  |  |  |  |  |  |  |
|  | **S_4_** |  | |  |  | **0.81 ± 0.05** | |  |  |  |  |  |  |  |  |
|  | **A_1_** |  | |  |  |  | | **0.68 ± 0.19** | **0.50 ± 0.04** | **0.47 ± 0.05** | **0.49 ± 0.05** |  |  |  |  |
|  | **A_2_** |  | |  |  |  | |  | **0.70 ± 0.14** | **0.59 ± 0.04** | **0.58 ± 0.04** |  |  |  |  |
|  | **A_3_** |  | |  |  |  | |  |  | **0.72 ± 0.11** | **0.65 ± 0.07** |  |  |  |  |
|  | **A_4_** |  | |  |  |  | |  |  |  | **0.73 ± 0.10** |  |  |  |  |
|  | **Bc_1_** |  | |  |  |  | |  |  |  |  | **0.60 ± 0.25** | **0.38 ± 0.01** | **0.41 ± 0.01** | **0.41 ± 0.04** |
|  | **Bc_2_** |  | |  |  |  | |  |  |  |  |  | **0.73 ± 0.13** | **0.63 ± 0.03** | **0.61 ± 0.05** |
|  | **Bc_3_** |  | |  |  |  | |  |  |  |  |  |  | **0.73 ± 0.11** | **0.64 ± 0.06** |
|  | **Bc_4_** |  | |  |  |  | |  |  |  |  |  |  |  | **0.70 ± 0.13** |
|  | **Average Set** | **0.73 ± 0.06** | | | | | **0.61 ± 0.08** | | | | | **0.58 ± 0.08** | | | |
|  |  |  | |  |  |  | | **Validation set** |  |  |  |  |  |  |  |
| Traits | **Tranning set** | **S_1_** | | **S_2_** | **S_3_** | **S_4_** | | **A_1_** | **A_2_** | **A_3_** | **A_4_** | **Bc_1_** | **Bc_2_** | **Bc_3_** | **Bc_4_** |
| d1 | **S_1_** | **0.65 ± 0.07** | | **0.57 ± 0.01** | **0.55 ± 0.01** | **0.55 ± 0.01** | |  |  |  |  |  |  |  |  |
|  | **S_2_** |  | | **0.74 ± 0.08** | **0.70 ± 0.05** | **0.70 ± 0.05** | |  |  |  |  |  |  |  |  |
|  | **S_3_** |  | |  | **0.80 ± 0.05** | **0.79 ± 0.05** | |  |  |  |  |  |  |  |  |
|  | **S_4_** |  | |  |  | **0.79 ± 0.06** | |  |  |  |  |  |  |  |  |
|  | **A_1_** |  | |  |  |  | | **0.56 ± 0.10** | **0.35 ± 0.05** | **0.34 ± 0.06** | **0.35 ± 0.02** |  |  |  |  |
|  | **A_2_** |  | |  |  |  | |  | **0.55 ± 0.10** | **0.40 ± 0.05** | **0.41 ± 0.03** |  |  |  |  |
|  | **A_3_** |  | |  |  |  | |  |  | **0.55 ± 0.15** | **0.43 ± 0.15** |  |  |  |  |
|  | **A_4_** |  | |  |  |  | |  |  |  | **0.55 ± 0.17** |  |  |  |  |
|  | **Bc_1_** |  | |  |  |  | |  |  |  |  | **0.49 ± 0.10** | **0.27 ± 0.02** | **0.31 ± 0.01** | **0.30 ± 0.06** |
|  | **Bc_2_** |  | |  |  |  | |  |  |  |  |  | **0.60 ± 0.19** | **0.44 ± 0.01** | **0.42 ± 0.04** |
|  | **Bc_3_** |  | |  |  |  | |  |  |  |  |  |  | **0.57 ± 0.10** | **0.43 ± 0.07** |
|  | **Bc_4_** |  | |  |  |  | |  |  |  |  |  |  |  | **0.59 ± 0.10** |
|  |  | **0.68 ± 0.04** | | | | | | **0.45 ± 0.10** | | | | **0.44 ± 0.09** | | | |

**S1 Table:** Reliability values of selection of generations advanced by self-pollination(S), random mating (A) and backcrossing (Bc) obtained from phenotyping and genotyping of the same generation (diagonally contemporary) or only from previous genotyping and phenotyping (outdated off-diagonally, horizontal reading) for the heritability trait equal to 0.30 in the scenario with an average degree of dominance equal to 0, 0.5 and 1.
