# Supplementary material for: Multigenerational prediction of genetic values using genome-enabled prediction
Source: PLoS One. 2019 Jan 17;14(1):e0210531. doi: 10.1371/journal.pone.0210531 (PMC6336252; doi:10.1371/journal.pone.0210531)
Supplement: S2 Table — (DOCX) [file pone.0210531.s002.docx]

**S2 Table**: Reliability values of selection of generations advanced by self-pollination and random mating (allogamous) obtained from phenotyping and genotyping of combined previous generations (multigenerational) or only from previous genotyping and phenotyping for heritability traits equal to 0.70, repeated in scenarios with an average degree of dominance level equal to 0, 0.5 and 1.

| Dominance | 0 | | | | 0.5 | | | | 1 | | | |
| --- | --- | --- | --- | --- | --- | --- | --- | --- | --- | --- | --- | --- |
| Multigerational Training | S_1_ | S_2_ | S_3_ | S_4_ | S_1_ | S_2_ | S_3_ | S_4_ | S_1_ | S_2_ | S_3_ | S_4_ |
| F_2_S_1_ | 0.89 ± 0.00 | 0.84 ± 0.00 | 0.82 ± 0.01 | 0.87 ± 0.01 | 0.84 ± 0.06 | 0.76 ± 0.06 | 0.74 ± 0.08 | 0.73 ± 0.07 | 0.75± 0.04 | 0,70 ± 0.01 | 0.70 ± 0.04 | 0.70 ± 0.04 |
| F_2_S_1_S_2_ |  | 0.90 ± 0.00 | 0.88 ± 0.00 | 0.81 ± 0.00 |  | 0.86 ± 0.06 | 0.90 ± 0.04 | 0.83 ± 0.06 |  | 0.82 ± 0.04 | 0.80 ± 0.04 | 0.80 ± 0.04 |
| F_2_S_1_S_2_S_3_ |  |  | 0.93 ± 0.00 | 0.92 ± 0.00 |  |  | 0.84 ± 0.08 | 0.88 ± 0.04 |  |  | 0.86 ± 0.03 | 0.85± 0.02 |
| Average Set | 0.87 ± 0.00 | | | | 0.82 ± 0.04 | | | | 0.77 ± 0.03 | | | |
| Multigerational Training | A1 | A2 | A3 | A4 | A1 | A2 | A3 | A4 | A1 | A2 | A3 | A4 |
| F_2_A_1_ | 0.92 ± 0.01 | 0.96 ± 0.01 | 0.93 ± 0.01 | 0.97 ± 0.11 | 0.76 ± 0.04 | 0.87 ± 0.02 | 0.77 ± 0.04 | 0.87 ± 0.02 | 0.49 ± 0.05 | 0.66 ± 0.05 | 0.52 ± 0.06 | 0.66 ± 0.04 |
| F_2_A_1_A_2_ |  | 0.91 ± 0.00 | 0.93 ± 0.01 | 0.93 ± 0.12 |  | 0.80 ± 0.06 | 0.82 ± 0.04 | 0.82 ± 0.04 |  | 0.57 ± 0.08 | 0.58 ± 0.06 | 0.59 ± 0.05 |
| F_2_A_1_A_2_A_3_ |  |  | 0.91 ± 0.00 | 0.92 ± 0.01 |  |  | 0.77 ± 0.04 | 0.77 ± 0.04 |  |  | 0.50 ± 0.06 | 0.50 ± 0.06 |
| Average Set | 0.92 ± 0.00 | | | | 0.80 ± 0.04 | | | | 0.56 ± 0.05 | | | |
| Multigerational Training | Bc_1_ | Bc _2_ | Bc _3_ | Bc _4_ | Bc_1_ | Bc _2_ | Bc _3_ | Bc _4_ | Bc_1_ | Bc_2_ | Bc_3_ | Bc_4_ |
| F_2_Bc_1_ | 0.88 ± 0.00 | 0.80 ± 0.02 | 0.80 ± 0.00 | 0.77 ± 0.00 | 0.80 ± 0.04 | 0.75 ± 0.02 | 0.75 ± 0.02 | 0.74 ± 0.03 | 0.71 ± 0.04 | 0.67 ± 0.01 | 0.68 ± 0.01 | 0.67 ± 0.00 |
| F_2_Bc_1_Bc_2_ |  | 0.90 ± 0.00 | 0.85 ± 0.00 | 0.83 ± 0.01 |  | 0.85 ± 0.02 | 0.80 ± 0.03 | 0.79 ± 0.03 |  | 0.76 ± 0.03 | 0.73 ± 0.02 | 0.71 ± 0.00 |
| F_2_Bc_1_Bc_2_Bc_3_ |  |  | 0.92 ± 0.00 | 0.87 ± 0.01 |  |  | 0.87 ± 0.03 | 0.83 ± 0.02 |  |  | 0.79 ± 0.03 | 0.74 ± 0.01 |
| Average Set | 0.85± 0.00 | | | | 0.72 ± 0.03 | | | | 0.72 ± 0.01 | | | |
